# Quality Over Quantity: Advantages of Using Alpha-Synuclein Preformed Fibril Triggered Synucleinopathy to Model Idiopathic Parkinson’s Disease

**DOI:** 10.3389/fnins.2018.00621

**Published:** 2018-09-04

**Authors:** Megan F. Duffy, Timothy J. Collier, Joseph R. Patterson, Christopher J. Kemp, D. Luke Fischer, Anna C. Stoll, Caryl E. Sortwell

**Affiliations:** ^1^Department of Translational Science and Molecular Medicine, Michigan State University, Grand Rapids, MI, United States; ^2^Mercy Health Hauenstein Neuroscience Medical Center, Grand Rapids, MI, United States

**Keywords:** Parkinson’s disease, alpha-synuclein, preformed fibrils, synucleinopathy, animal models

## Abstract

Animal models have significantly advanced our understanding of Parkinson’s disease (PD). Alpha-synuclein (α-syn) has taken center stage due to its genetic connection to familial PD and localization to Lewy bodies, one pathological hallmark of PD. Animal models developed on the premise of elevated alpha-synuclein via germline manipulation or viral vector-mediated overexpression are used to investigate PD pathophysiology and vet novel therapeutics. While these models represented a step forward compared to their neurotoxicant model predecessors, they rely on overexpression of supraphysiological levels of α-syn to trigger toxicity. However, whereas *SNCA-*linked familial PD is associated with elevated α-syn, elevated α-syn is not associated with idiopathic PD. Therefore, the defining feature of the α-syn overexpression models may fail to appropriately model idiopathic PD. In the last several years a new model has been developed in which α-syn preformed fibrils are injected intrastriatally and trigger normal endogenous levels of α-syn to misfold and accumulate into Lewy body-like inclusions. Following a defined period of inclusion accumulation, distinct phases of neuroinflammation and progressive degeneration can be detected in the nigrostriatal system. In this perspective, we highlight the fact that levels of α-syn achieved in overexpression models generally exceed those observed in idiopathic and even *SNCA* multiplication-linked PD. This raises the possibility that supraphysiological α-syn expression may drive pathophysiological mechanisms not relevant to idiopathic PD. We argue in this perspective that synucleinopathy triggered to form within the context of normal α-syn expression represents a more faithful animal model of idiopathic PD when examining the role of neuroinflammation or the relationship between a-syn aggregation and toxicity.

## Introduction

Parkinson’s disease (PD) is the second most common neurodegenerative disorder, affecting 7–10 million individuals worldwide. Though PD was first described over 200 years ago by James Parkinson, no therapies currently exist to halt or slow nigrostriatal degeneration despite scores of preclinical studies predicting the success of particular disease-modifying treatment strategies. This “translational abyss" may be due in part to the failure of animal models to faithfully recapitulate human disease and, more specifically, failure to use the appropriate PD animal model.

During the past two decades, numerous preclinical studies have used overexpression of human wildtype or mutant alpha-synuclein (α-syn) in *Drosophila*, rodents and non-human primates to model PD. Overexpression is achieved in these models via transgenic engineering or via injection of viral vectors. However, while these α-syn models represented an advance over neurotoxicant models through the incorporation of α-syn, the mechanism of toxicity and the neuropathology generated differ from idiopathic PD in key respects. Specifically, α-syn overexpression paradigms result in α-syn protein expression levels that far exceed levels associated with idiopathic PD, levels which can induce exacerbated neuroinflammation. Further, viral vector-mediated α-syn overexpression results in pathology in limited circuitry and can lack a protracted phase of classical Lewy body-like inclusion pathology. We contend that these shortcomings in the α-syn overexpression-based models have hindered our understanding of the pathogenic contributions of normal endogenous α-syn in idiopathic PD and have handicapped our ability to predict efficacy of novel neuroprotective therapeutics.

Recently, a model of synucleinopathy and nigral degeneration induced by intracerebral administration of sonicated preformed fibrils of α-syn (PFFs) has provided a new platform to study the pathogenic cascade in which normal levels of endogenous α-syn levels are triggered to misfold, template and accumulate. The engagement of physiologic levels of endogenous α-syn in the α-syn PFF model allows for the assessment of differential neural circuit vulnerabilities to α-syn inclusion formation and toxicity. The α-syn PFF model recapitulates many features of human idiopathic PD, namely: a protracted interval of accumulation of insoluble Lewy-like pathology, α-syn inclusion triggered neuroinflammation and degeneration of specific neuronal subpopulations.

Comprehensive comparisons of the viral vector mediated a-syn overexpression model and the a-syn PFF model have been previously conducted (Volpicelli-Daley et al., 2016; Koprich et al., 2017). In this perspective, we highlight pathological features of human idiopathic and *SNCA*-linked familial PD in order to compare these features to those observed in the α-syn PFF model. Ultimately, these sporadic PD and α-syn PFF model features will be placed in the context of the pathology induced by overexpression of α-syn, with a focus on viral vector-mediated α-syn overexpression. We argue the perspective that the synucleinopathy and the inclusion-initiated nigral degeneration resulting from α-syn PFF injection provides a superior platform for comprehending the pathophysiology of idiopathic PD and by extension, a superior platform for evaluating potential neuroprotective therapeutics.

### Parkinson’s Disease

#### Alpha-Synuclein (α-Syn) Expression and Localization in Idiopathic and SNCA-Linked Familial PD

While the majority (90–95%) of PD cases do not have an identified genetic component, they share with familial PD a role for the protein α-syn. In the CNS, α-syn accounts for approximately 1% of total protein and is localized primarily in the cytosol of axon terminals, with known roles in membrane bending, synaptic transmission, and plasticity (Bendor et al., 2013; Benskey et al., 2016). In 1997 it was first revealed that an Ala53Thr (A53T) substitution in the α-syn gene (*SNCA*) was associated with young onset, autosomal dominant PD (Polymeropoulos, 1998). Shortly thereafter it was shown in subjects with idiopathic PD that fibrillar α-syn was a major component of Lewy bodies (Spillantini et al., 1998). Subsequent work identified additional point mutations [A30P, E46K, H50Q, and G51D; (Kruger et al., 1998; Somme et al., 2011; Appel-Cresswell et al., 2013; Lesage et al., 2013)] that increase the risk of PD. The effects of these missense mutations on α-syn protein conformational state, solubility, membrane association, and aggregation kinetics have been extensively studied *in vitro* and *in vivo* (Jo et al., 2000; Khalaf et al., 2014; Dettmer et al., 2015; Xu et al., 2016). In addition to point mutations in *SNCA*, duplications or triplications of wildtype *SNCA* are also causative in familial PD. Collectively, this body of work provides compelling evidence that α-syn plays a central role in the pathophysiology of both familial and idiopathic PD.

As expected, studies of α-syn multiplication carriers demonstrate that α-syn mRNA and protein are elevated in these genetic forms of the disease with 1.5–2-fold increases in abundance reported (Singleton et al., 2003; Chartier-Harlin et al., 2004; Farrer et al., 2004; Fuchs et al., 2007; Olgiati et al., 2015) Notably, *SNCA* triplication carriers exhibit earlier onset and more-rapidly progressing PD compared to duplication carriers or sporadic PD patients, further lending support that dose-dependent increases in α-syn are a driving factor in disease onset and severity. However, in idiopathic PD the evidence for either elevated α-syn mRNA or α-syn protein is lacking. Analysis of α-syn mRNA levels within individual nigral neurons from early or late idiopathic PD subjects has revealed conflicting results with no differences reported (Tan et al., 2005; Su et al., 2017), increases reported (Grundemann et al., 2008) or decreases reported (Neystat et al., 1999; Kingsbury et al., 2004). Analysis of total α-syn protein levels in post-mortem idiopathic PD tissue suggests either a modest transient increase or similar levels to age-matched control (Zhou et al., 2011). In contrast to *SNCA-*linked familial PD, the concept that increases in α-syn levels drive pathophysiology in idiopathic PD is less supported.

Whereas total expression levels of α-syn appear to distinguish *SNCA* multiplication carriers from idiopathic PD patients, changes in the solubility, membrane association, and abundance of post-translationally modified forms α-syn are similar in both patient subgroups. Investigations beyond a focus on α-syn abundance have identified changes in cellular localization and post-translational modifications as potential mechanisms of α-syn-mediated toxicity, as reviewed in Jo et al. (2000), Lee et al. (2002), Auluck et al. (2010), Tong et al. (2010), van Rooijen et al. (2010), Barrett and Timothy Greenamyre (2015), Oueslati (2016), and Burre et al. (2018). In idiopathic PD, studies have consistently demonstrated shifts in the ratio of soluble to insoluble α-syn without concurrent changes in total α-syn levels. Specifically: decreases in soluble monomeric α-syn with concurrent increases in soluble phosphorylated α-syn (pSyn) along with increases in membrane-bound α-syn have been observed in particularly vulnerable regions (SN and cortex) in sporadic PD cases (Gibb and Lees, 1988; Irizarry et al., 1998). Similar observations have also been made in samples derived from *SNCA* triplication carriers, albeit with increased magnitude and less regional specificity (Tong et al., 2010). The fact that both genetic and idiopathic forms of PD are associated with α-syn phosphorylation and increased membrane interactions suggests a role for these phenomena in PD pathophysiology.

#### Development of Lewy Pathology and Affected Circuitry

Confirmed diagnosis of PD is not made until Lewy bodies (LBs) and Lewy neurites (LNs) are observed upon post-mortem evaluation. LBs are composed of dozens of proteins, which may include α-syn, neurofilament, p62 and ubiquitin. pSyn staining is the most common immunohistochemical method of LB detection in post-mortem tissue, however, it should be noted that pSyn inclusions likely represent end stage LB development. Immature LBs, termed “pale bodies” are more often observed in early disease stages. Pale bodies are strongly immunoreactive for α-syn and manifest as intracellular diffuse, granular eosinophilic material with ill-defined borders. As disease stage advances, mature cytoplasmic LBs predominate over pale bodies and differ slightly in appearance depending on location in the cortex or brainstem (Gibb and Lees, 1988; Irizarry et al., 1998; Stefanis, 2012). With increasing maturity, LB and LN inclusions display a dense core with radiating filaments, and are strongly Thioflavin-S positive for beta-sheet structure and resistant to digestion by proteinase-K (Neumann et al., 2002; Li et al., 2010).

Although Lewy pathology is widespread in PD brain, it occurs in well-defined regions including the substantia nigra pars compacta, amygdala, olfactory bulb, temporal, frontal and parietal cortices (Halliday et al., 2011). Braak and colleagues developed a staging scheme for PD based on the location of pSyn LB and LN inclusions. Braak proposed that inclusions are first found in the olfactory bulb and dorsal motor nucleus of the vagus nerve, and follow an ascending pattern through the brainstem and finally the cortex (Braak et al., 2003; Dickson, 2018), lending to the debate of PD as a prion-like disease (Braak et al., 2003; Halliday et al., 2011; van de Berg et al., 2012). However, it should be noted that ∼50% of cases do not follow this staging scheme (Burke et al., 2008; Jellinger, 2008; Kalaitzakis et al., 2008; Beach et al., 2009). Other theories suggest parallel rather than stepwise accumulation of Lewy pathology based on differential vulnerability profiles of various cell types and regions (Engelender and Isacson, 2017).

#### Neuroinflammation

In recent years, neuroinflammation has been proposed as a contributor to neurodegeneration in PD and a potential target for disease modification. Early observations of post-mortem tissue describe a local increase in inflammatory markers in the SN associated with microglia, notably human-leukocyte antigen-D related [HLA-DR; (McGeer et al., 1988a,b, 1993; Imamura et al., 2003)], the human analog for major histocompatibility complex-II (MHC-II; antigen presentation). Not only has increased MHC-II expression been observed, it correlates positively with α-syn burden (Croisier et al., 2005). More recent work has implicated mutations in HLA-DR in amplified risk for developing PD, and levels of MHC-II are increased in cases of Incidental Lewy Body Disease [Braak stage I-II; (Dijkstra et al., 2015)] suggesting that inflammation may be at least in part, a contributing factor to ongoing degeneration (Kannarkat et al., 2015). On a broad level, cytokine measurements from patient biofluids (plasma and CSF) have consistently shown deviations from normal proinflammatory and anti-inflammatory cytokine levels compared to controls, although results are conflicting and may stem from variance in subject disease duration and time of sample collection (Mogi et al., 1996; Lindqvist et al., 2013; Eidson et al., 2017). However, while studies of patient tissue and biofluids have suggested that inflammation is involved in PD, these samples represent a single point in time over long disease duration. It is unclear whether neuroinflammatory markers that associate with LBs precede the formation of LBs and if the LB-containing neurons ultimately degenerate. Thus, the time-course of neuroinflammation in relation to α-syn accumulation and aggregation and nigral degeneration in human PD has yet to be determined.

### Using the α-Syn PFF Seeded Synucleinopathy to Model Idiopathic PD

#### PFF-Induced Synucleinopathy in the Context of Normal Levels of Endogenous α-Syn

Given that idiopathic PD is not associated with an increase in total α-syn protein levels, synucleinopathy that arises in the context of normal endogenous α-syn levels would more faithfully recapitulate this key characteristic of the non-genetic form of PD. In contrast, previous models have relied on global overexpression (transgenics) or targeted overexpression (viral vector-mediated) of α-syn. The α-syn preformed fibril (PFF) model represents an approach in which synucleinopathy is induced in an environment of normal α-syn protein levels. The PFF model was first developed *in vitro* by introduction of α-syn PFFs to primary neuronal cultures. Briefly, α-syn fibrils are generated from recombinant α-syn monomers and sonicated to form smaller ∼50 nm fragments which are introduced to cell culture (Volpicelli-Daley et al., 2011). The PFFs are internalized by neurons, template and recruit endogenous α-syn and accumulate as inclusions of insoluble pSyn (Luk et al., 2009; Volpicelli-Daley et al., 2011, 2014). The pSyn inclusions ultimately lead to neuronal dysfunction and degeneration. This toxicity is not due to introduction of the high quantity of PFFs *per se* but can be directly linked to the recruitment of endogenous α-syn into inclusions as evidenced by the fact that PFFs do not induce toxicity when applied to α-syn^−/−^ primary neurons (Volpicelli-Daley et al., 2011; Luk et al., 2012b).

#### Development of Abundant Lewy-Like Pathology in Multiple Extra Nigrostriatal Regions

**FIGURE 1:**
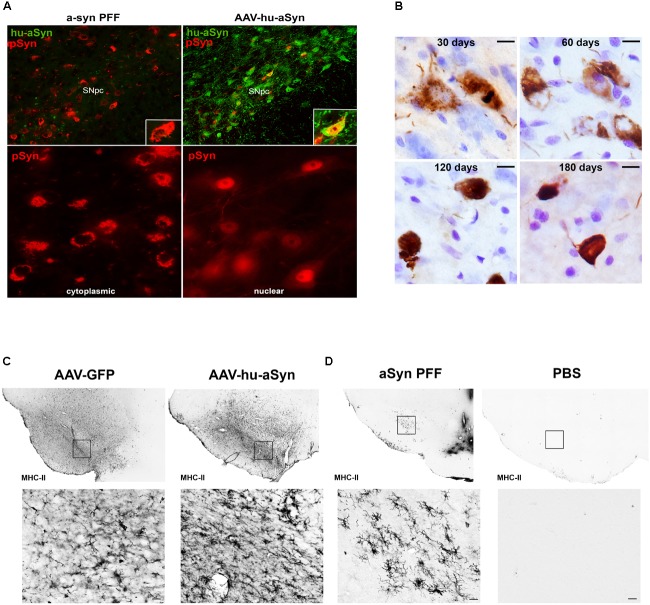
Comparison of alpha-synuclein pathology and inflammation between AAV-overexpression and α-syn preformed fibril models. **(A)** Intrastriatal injection of α-syn PFFs results in accumulation of phosphorylated α-syn (pSyn, red) inclusions in the cytoplasm of SNc neurons, similar to cytoplasmic Lewy bodies in PD. Intranigral delivery of rAAV2/5 expressing human wildtype α-syn results in transduction of many nigral neurons with abundant human α-syn (green), but relatively few pSyn inclusions (red) in the cytoplasm in comparison to PFF-induced pSyn inclusions number. pSyn inclusions induced by AAV-mediated overexpression of α-syn are predominantly nuclear. **(B)** Evolution of Lewy body-like pSyn inclusion formation in the substantia nigra pars compacta induced by intrastriatal PFF injection. Inclusions follow a similar pattern to that observed in human PD, with immature inclusions appearing diffuse and granular and becoming more dense and compact over time. **(C,D)** Comparison of MHC-II expression 8 weeks following intranigral rAAV2/5 GFP or human wildtype α-syn vector injection **(C)** or intrastriatal injection of sonicated preformed murine α-syn fibrils or PBS **(D)**. In all cases no loss of nigral dopamine neurons was observed. Injection of AAV2/5 GFP or human α-syn is associated with robust increases in MHC-II on microglia throughout the mesencephalon, suggesting that the inflammation observed is largely attributable to surgical injection at the transduction site. In contrast, intrastriatal injection of PFFs results in MHC-II expression more localized to the SNc, with few to no MHC-II immunoreactive microglia evident in PBS injected animals. Abbreviations: rAAV2/5, recombinant adeno-associated virus 2/5; α-syn, alpha-synuclein; GFP, green fluorescent protein; MHC-II, major histocompatibility complex-II; PBS, phosphate buffered saline; PFFs, alpha-synuclein preformed fibrils; pSyn, alpha-synuclein phosphorylated at serine 129; SNc, substantia nigra pars compacta.

The α-syn PFF model has since been extended to wildtype mice (Luk et al., 2012a) and rats (Paumier et al., 2015; Abdelmotilib et al., 2017; Duffy et al., 2018) and most recently non-human primates (Shimozawa et al., 2017). These studies demonstrate that direct intracerebral injection of α-syn PFFs leads to accumulation of insoluble pSyn inclusions resembling Lewy pathology, all taking place in an environment of normal endogenous α-syn expression levels. Similar to *in vitro* findings, the injection of the high quantity of PFFs in a-syn^−^/^−^ mice does not result in accumulation formation or degeneration (Luk et al., 2012b), illustrating the contribution and requirement of normal endogenous a-syn levels in the development of the pathological cascade. The similarity between α-syn PFF induced pSyn inclusions and human Lewy pathology in the SNc is particularly striking. At early time points post-injection, nigral pSyn immunoreactive inclusions resemble pale bodies: granular, diffuse and cytoplasmic (**Figure 1B**), whereas cortical inclusions occur abundantly in neurites and appear tendril-like in the soma. Over time, pSyn inclusions condense into more compact aggregates. The observed pSyn inclusions frequently colocalize with markers commonly observed in human LBs including p62 and ubiquitin, consist of α-syn oligomers and fibrils, and are also Thioflavin-S positive and proteinase-K resistant (Paumier et al., 2015; Duffy et al., 2018). Two photon microscopy has confirmed that neurons that form these pSyn inclusions ultimately degenerate (Osterberg Valerie et al., 2015). Similarly, the magnitude of pSyn inclusion formation observed in the substantia nigra 2 months following PFF injection can be used to predict the ultimate extent of nigral degeneration observed at 6 months (Duffy et al., 2018). The protracted course of these aggregation and degeneration events provides investigators the ability to focus on particular phases of the synucleinopathy cascade.

*In vivo*, the spatial emergence of LB and LN-like pathology is dependent on the location of PFF injection. For example, several groups have demonstrated that intrastriatal injections of PFFs result in pSyn inclusion accumulation in cell bodies of regions innervating the striatum (Wall et al., 2013): namely the substantia nigra, agranular insular and motor cortices, and amygdala (Luk et al., 2012a,b; Paumier et al., 2015; Abdelmotilib et al., 2017; Duffy et al., 2018), suggesting that axon terminals internalize the PFFs. The occurrence of pathology in these defined regions supports the concept of retrograde transport and templating of endogenous α-syn within neurons exposed to the injection site, rather than prion-like spread throughout the extensive brain networks. Importantly, areas in which pathology is observed are implicated in human PD (Halliday et al., 2011).

#### Neuroinflammation

In addition to generating α-syn pathology in multiple brain regions, intrastriatal injection of α-syn PFFs allows for investigations to delineate the time course of the inflammatory response in the substantia nigra. Due to the fact that the injection site (striatum) is spatially separated from the substantia nigra, inflammation related to surgical injection is minimized. Intrastriatal injection of PFFs, but not vehicle or other protein controls, induces peak reactive **Figure 1D** microglial morphology in the substantia nigra at 2 months, corresponding to the time point in which the greatest number of pSyn inclusions are observed (Duffy et al., 2018). In addition, a pSyn specific increase in MHC-II immunoreactive microglia is observed at this same time point, significantly correlating with pSyn inclusion load. This relationship between MHC-II immunoreactivity and α-syn inclusion burden is reminiscent to what has been reported in idiopathic PD (Croisier et al., 2005) providing face validity to the α-syn PFF model. However, while MHC-II is classically associated with a proinflammatory phenotype, it is unknown whether MHC-IIir microglia play a direct role in neurodegeneration, as a recent study has suggested that decreased expression of MHC-II and its regulator, *Mhc2ta*, augment α-syn pathology and accelerate degeneration (Jimenez-Ferrer et al., 2017). In addition, determining the stimulus of MHC-II expression is warranted (i.e., classical stimuli such as extracellular proteins or alternative induction by IFN-γ secretion). It is possible that the gradual and modest nature of degeneration is insufficient to activate a sufficient immune response, rendering microglia as neutral. In addition, microglia promote tissue healing and repair, as they represent the brain’s primary defense system against insult and infection. Therefore, clarifying the beneficial or detrimental role[s] of microglia at different disease stages is warranted. We suggest that the PFF model provides an improved platform for clarifying the role of microglia in synucleinopathy and degeneration, as it avoids the confounds of surgical artifact, compressed time-course, and irrelevant inflammatory responses resulting from continuous supraphysiological expression of human protein.

### Is α-Syn Overexpression Analogous to Idiopathic PD?

#### Reliance on Supraphysiological α-Syn Levels and Lack of Protracted Lewy-Like Pathology

The development of AAV-α-syn overexpression models represents a significant advantage over previous neurotoxicant models which lack synucleinopathy as a component of disease pathology. Notably, AAV-α-syn overexpression models have provided insights into direct mechanisms of α-syn toxicity, including axonal and dopamine transport deficits and impairment of protein degradation pathways (Chung et al., 2009; Gaugler et al., 2012; Volpicelli-Daley et al., 2016; Koprich et al., 2017). Models induced by intranigral injection of adeno-associated (AAV) or lentiviral (LV) overexpressing human wildtype or mutant α-syn often result in dramatic increases in the levels of protein (Kirik et al., 2002; Klein et al., 2002; Lo Bianco et al., 2002; Yamada et al., 2004; Ulusoy et al., 2010; Lundblad et al., 2012; Mulcahy et al., 2012; Oliveras-Salvá et al., 2013; Van der Perren et al., 2015; Ip et al., 2017). Although final protein levels are titer dependent, many studies report levels of α-syn 2–20× higher than normal endogenous expression levels, as reviewed extensively (Volpicelli-Daley et al., 2016). As these levels far exceed those observed in either idiopathic, duplication and even SNCA triplication carriers, they raise the potential for pathophysiological mechanisms specific to supraphysiological α-syn expression, mechanisms that may not be relevant to idiopathic PD. Indeed, the downregulation of multiple trophic factor responsive genes is observed with fourfold, but not lower α-syn overexpression levels (Decressac et al., 2012; Su et al., 2017). *In vitro* studies show that following transduction, neurons release multiple forms of α-syn (Kim et al., 2013). It is therefore likely that α-syn is similarly released from neurons transduced *in vivo*. It is unclear to what extent inclusion-bearing neurons release α-syn, if at all.

Lewy pathology in PD is widespread and likely develops in multiple regions concurrently. In contrast, AAV and LV-mediated overexpression models drive α-syn expression in discrete circuitries, most often the nigrostriatal system. Another consideration is the form of Lewy pathology generated by α-syn overexpression. Although pSyn immunoreactive and proteinase-K resistant inclusions have been reported they are most often small and punctate (Ip et al., 2017) in contrast to the large, cytoplasmic aggregates seen in human PD and in the α-syn PFF model. Further, frequently the pSyn inclusions generated following α-syn overexpression are localized to the nucleus **Figure 1A** and **Supplementary Material**, unlike the cytoplasmic LBs that are the hallmark of PD and are observed in the PFF model (**Figure 1B**). A dramatic rise in cytosolic pSyn has been documented in PD (Zhou et al., 2011), and thus pSyn localized to the nucleus that is observed with viral vector-mediated α-syn overexpression would prevent pSyn’s ability to interact with cytoplasmic proteins and structures.

Transgenic models overexpressing wildtype or mutant α-syn consistently display widespread synuclein pathology including proteinase-K resistant inclusions and behavioral deficits (Tanji et al., 2010; Yamakado et al., 2012). While there are some exceptions (Nuber et al., 2013), the majority of transgenic models do not exhibit robust, protracted nigral degeneration (Matsuoka et al., 2001; Fernagut and Chesselet, 2004). Thus, while transgenic models are adequate for investigating development of Lewy-like pathology as a consequence of germline genetic changes, most fail to recapitulate downstream nigral degeneration and thus have limited translational potential for evaluation of neuroprotective strategies.

#### Inflammation in AAV and LV-Mediated Overexpression Models: Location, Location, Location

Another feature of viral vector-mediated α-syn overexpression models that is often leveraged is their ability to produce a robust neuroinflammatory response as indicated by microgliosis, MHC-II and CD68 on microglia, in addition to production of proinflammatory cytokines (Chung et al., 2009; Sanchez-Guajardo et al., 2010; Harms et al., 2013). However, there are several considerations when interpreting the disease relevance of the neuroinflammatory response in this paradigm. First, the majority of viral vector models are induced by direct intranigral injection which alone, in the absence of α-syn overexpression, can trigger a pronounced increase in MHC-II immunoreactive microglia (**Figure 1C**). This suggests that a significant component of the inflammatory response resulting from intranigral injections of α-syn vectors is due to the injection itself. Further, as previously stated, α-syn overexpression paradigms can result in the release of supraphysiological levels of α-syn into the immediate environment. This secretion of α-syn likely triggers a neuroinflammatory response in the absence of degeneration (**Figure 1C**) that has little to do with the disease state attempting to be modeled. Lastly, the majority of AAV and LV models overexpress human α-syn, not rodent α-syn, in rats and mice (Fischer et al., 2016; Volpicelli-Daley et al., 2016). As rat and mouse α-syn differs from human α-syn by eight amino acids, it is plausible that overexpression of the foreign human protein may initiate an artificial inflammatory response.

## Conclusion

The multiple features of the α-syn PFF model and the AAV a-syn model have been extensively reviewed elsewhere (refs). However, we contend that some of these model-specific features deserve specific attention when modeling idiopathic PD (**Table 1**). In particular, we offer that that α-syn PFF model may be better suited for studies of neuroinflammation and the relationship between α-syn aggregation and toxicity in idiopathic PD. The ability to advance our understanding of pathophysiology in idiopathic PD and predict the efficacy of novel therapeutics is dependent on the fidelity of animal models to the disease state. When modeling idiopathic PD, the presence of LB-like α-syn inclusions within the context of normal endogenous α-syn levels in multiple brain regions, which ultimately results in progressive nigrostriatal degeneration are an essential model feature. While α-syn overexpression models have advanced our understanding of α-syn-mediated toxicity, they depend on focal expression of supraphysiological levels of α-syn in a limited circuitry. In contrast to *SNCA-*linked familial PD, clinicopathologic evidence does not support the concept that increases in α-syn levels drive pathophysiology in idiopathic PD and therefore α-syn overexpression may trigger pathogenic mechanisms that may not be relevant to idiopathic PD. We propose that accumulation of Lewy body-like inclusions in multiple regions induced by injection of PFFs in the context of normal α-syn levels, ultimately resulting in downstream inflammation and progressive nigral degeneration, more faithfully models the sequence of events in idiopathic PD. Additionally, findings from the PFF-model may be applicable to other synucleinopathies. Thus, the synucleinopathy induced by α-syn PFF injections represents an exceptional preclinical PD model to investigate the pathogenic contribution of endogenous α-syn, and assess novel disease-modifying therapeutics.

**Table 1:** Considerations when modeling idiopathic PD in rodents.

Feature	AAV-overexpression models	α-syn PFF model
**Ability to examine impact of α-synuclein inclusions distinct from degeneration**	**Difficult**	**Straightforward**
	Simultaneous α-syn overexpression and aggregation progresses rapidly to degeneration over the course of weeks	Distinct interval of inclusion formation followed by degeneration over a protracted time course
**Injection artifact**	**Confound**	**Less of a factor**
	Direct injections into the SN produce marked neuroinflammatory response that can make interpretation difficult	Direct injections into the striatum have less of an impact within the SN
**α-synuclein levels**	**Not analogous to idiopathic PD**	**Normal endogenous α-syn levels**
	Continuous supraphysiological α-syn levels produced by forced overexpression are not analogous to idiopathic PD	Pathophysiology results from templating of normal levels of α-syn
**Extranigral α-synuclein pathology**	**Not present**	**α-syn pathology in multiple regions**
	Pathology limited to the nigrostriatal system	Allows for the examination of events outside of nigrostriatal system

*Abbreviations: AAV, adeno-associated virus; α-syn, alpha-synuclein; PD, Parkinson’s disease; PFFs, alpha-synuclein preformed fibrils; SN, substantia nigra.*

## Ethics Statement

All procedures performed in studies involving animals were in accordance with the ethical standards of the Institute for Animal Use and Care Committee (IACUC) at Michigan State University.

## Author Contributions

This manuscript was conceived and organized by MD, TC, and CS and discussed among all authors. Data were generated by MD, CK, CS, DF, and AS. The manuscript was first written and revised by MD and CS, and it was reviewed and critiqued by all authors.

## Conflict of Interest Statement

The authors declare that the research was conducted in the absence of any commercial or financial relationships that could be construed as a potential conflict of interest.
