# Supplementary material for: Quality Over Quantity: Advantages of Using Alpha-Synuclein Preformed Fibril Triggered Synucleinopathy to Model Idiopathic Parkinson’s Disease
Source: Front Neurosci. 2018 Sep 4;12:621. doi: 10.3389/fnins.2018.00621 (PMC6132025; doi:10.3389/fnins.2018.00621)
Supplement: Supplementary file 1 [file Data_Sheet_1.docx]

**Supplementary Methods:**

*Vector construction and intranigral injection*

Recombinant adeno-associated viral vector pseudotype 2/5 (rAAV2/5) was used to overexpress human wildtype α-syn (rAAV-hu-α-syn) or green fluorescent protein (GFP) and constructed as previously described [91, 92], resulting in a final titer of 1.8 x 10^12^ genome copies per ml [92] or 1.8 x 10^13^ genome copies per ml [91], respectively. Male rats received two unilateral nigral injections of rAAV2/5-hu-α-syn (4μl total; AP −5.3 mm, ML + 2.0 mm, DV −7.2 mm and AP −6.0 mm, ML + 2.0 mm, DV −7.2 mm relative to dura) or rAAV2/5-GFP at a rate of 0.5µl/minute, 2μl per site. Animals were euthanized 8 weeks following injection as described below.

*PFF Generation and Intrastriatal Injection*

Purification of recombinant, full-length mouse α-syn and in *vitro* fibril assembly and intrastriatal injections were performed as previously described [58, 61, 63]. Prior to sonication, α-syn fibrils were assessed to verify lack of contamination (LAL Assay, (~1 Endotoxin Units /mg), presence of high molecular weight species (sedimentation assay), beta sheet conformation (Thioflavin T) and structure (transmission electron microscopy). Prior to injection, PFFs were thawed, diluted in sterile Dulbecco’s PBS (DPBS, 2μg/μl) and sonicated at room temperature using an ultrasonicating homogenizer (300VT; Biologics, Inc., Manassas, VA) with the pulser set at 20%, power output at 30% for 60 pulses at 1 second each. Sonicated PFFs were kept at room temperature throughout the duration of the surgical procedure. Male, 2-month old rats were deeply anesthetized with isoflurane and received two 2μl unilateral intrastriatal injections (4μl total; AP +1.6, ML +2.4, DV -4.2; AP -1.4, ML +2.0, DV -7.0 from skull) either of sonicated mouse α-syn PFFs (2μg/μl as described previously) or an equal volume of DPBS at a rate at 0.5µl/minute. Injections were administered made using a pulled glass needle attached to a 10 µl Hamilton syringe. After each injection, the needle was left in place for 1 minute, retracted 0.5 mm, left in place for an additional 2 minutes and then slowly withdrawn. Animals were monitored post-surgery and euthanized at monthly intervals up to 6 months.

*Immunohistochemistry*

All animals were euthanized via pentobarbital overdose (60mg/kg) and intracardially perfused with heparinized 0.9% saline followed by cold 4% paraformaldehyde in 0.1M PO_4_. Brains were extracted and post-fixed in 4% PFA for 48 hours and placed in 30% sucrose until sunk. Brains were frozen on a sliding microtome and cut at 40μm. Free-floating sections (1:6 series) were transferred to 0.1M tris buffered saline (TBS). Following washes, endogenous peroxidases were quenched in 3% H_2_O_2_ for 1 hour and rinsed in TBS. Sections were blocked in 10% normal goat serum/0.5% Triton-X 100 in TBS (NGS, Gibco; Tx-100 Fischer Scientific) for 1 hour. Following block, sections were labeled with the following primary antibodies: mouse anti-human α-syn (transduction verification; Invitrogen, Carlsbad, CA; AHB0261; 1:2000; [91]), mouse anti-phosphorylated α-syn at Serine 129 (pSyn, 81A; Abcam, Cambridge, MA; AB184674; 1:10,000; [63]), or mouse anti-rat major histocompatibility complex-II for antigen presenting microglia (MHC Class II RT1B clone OX-6; BioRad, Hercules, CA; MCA46G, 1:5000; [63]) overnight at 4ºC. Following washes, sections were incubated in biotinylated secondary antibodies (1:500) against mouse IgG (Millipore, Temecula, CA; AP124B) followed by washes in TBS and 2-hour incubation with Vector ABC standard detection kit (Vector Laboratories, Burlingame, CA; PK-6100). Labeling for pSyn and MHC-II was visualized by development in 0.5 mg/ml 3,3’ diaminobenzidine (DAB; Sigma-Aldrich St. Louis, MO; D5637-10G) and 0.03% H_2_O_2_. Slides were dehydrated in ascending ethanol series then xylenes before coverslipping with Cytoseal (Richard-Allan Scientific, Waltham, MA) and imaged on a Nikon Eclipse 90i microscope with a QICAM camera (QImaging, Surrey, British Colombia, Canada) and Nikon Elements AR (version 4.50.00, Melville, NY).

*Immunofluorescence*

Free-floating sections (1:6 series) were transferred to 0.1M tris buffered saline (TBS) and washed. Sections were blocked in 10% normal goat serum/0.5% Triton-X 100 in TBS (NGS, Gibco; Tx-100 Fischer Scientific) for 1 hour. Following block, sections were labeled with mouse anti-human α-syn (IgG1; Invitrogen AHB0261; 1:2000) and mouse anti-phosphorylated α-syn (pSyn, 81A; IgG2a; Abcam, Cambridge, MA; AB184674; 1:10,000) overnight at 4ºC. Sections were washed and incubated in secondary antibodies goat anti-mouse against 81A IgG2A (Invitrogen; Carlsbad, CA; A-21135; Alexa Fluor 594) and goat anti-mouse IgG1 against a-syn (Invitrogen; Carlsbad, CA; A-21121; Alexa Fluor 488) at 1:500 in 1% NGS/0.5% Tx-100 for 2 hours. Sections were washed, mounted on subbed slides and coverslipped with VectaShield hardset mounting media (Vector Labs; H1400) and visualized on a on a Nikon Eclipse 90i microscope with a QICAM camera (QImaging, Surrey, British Colombia, Canada) and Nikon Elements AR (version 4.50.00, Melville, NY).
